# Selective Boosting of CCR7-Acting Chemokines; Short Peptides Boost Chemokines with Short Basic Tails, Longer Peptides Boost Chemokines with Long Basic Tails

**DOI:** 10.3390/ijms23031397

**Published:** 2022-01-26

**Authors:** Emma Probst Brandum, Astrid Sissel Jørgensen, Marina Barrio Calvo, Katja Spiess, Francis C. Peterson, Zhang Yang, Brian F. Volkman, Christopher T. Veldkamp, Mette Marie Rosenkilde, Christoffer Knak Goth, Gertrud Malene Hjortø

**Affiliations:** 1 Department of Biomedical Sciences, University of Copenhagen, 2200 Copenhagen, Denmark; emma.brandum@sund.ku.dk (E.P.B.); asj@sund.ku.dk (A.S.J.); ktsp@ssi.dk (K.S.); rosenkilde@sund.ku.dk (M.M.R.); goth@sund.ku.dk (C.K.G.); 2 Evaxion Biotech A/S, 2970 Horsholm, Denmark; mbc@evaxion-biotech.com; 3 Virus and Microbiological Special Diagnostics, Statens Serum Institut, 2300 Copenhagen, Denmark; 4 Department of Biochemistry, Medical College of Wisconsin, Milwaukee, WI 53226, USA; fpeterso@mcw.edu (F.C.P.); bvolkman@mcw.edu (B.F.V.); 5 Copenhagen Center for Glycomics, University of Copenhagen, Noerregade 10, 1165 Copenhagen, Denmark; yang@sund.ku.dk; 6 Department of Chemistry, University of Wisconsin-Whitewater, Whitewater, WI 53190, USA; veldkamc@uww.edu

**Keywords:** basic peptide, CCR7, biased signaling, CCL21, CCL19

## Abstract

The chemokine receptor CCR7 and its ligands CCL19 and CCL21 regulate the lymph node homing of dendritic cells and naïve T-cells and the following induction of a motile DC-T cell priming state. Although CCL19 and CCL21 bind CCR7 with similar affinities, CCL21 is a weak agonist compared to CCL19. Using a chimeric chemokine, CCL19^CCL21N-term|C-term^, harboring the N-terminus and the C-terminus of CCL21 attached to the core domain of CCL19, we show that these parts of CCL21 act in a synergistic manner to lower ligand potency and determine the way CCL21 engages with CCR7. We have published that a naturally occurring basic C-terminal fragment of CCL21 (C21TP) boosts the signaling of both CCL19 and CCL21. Boosting occurs as a direct consequence of C21TP binding to the CCR7 N-terminus, which seems to free chemokines with basic C-termini from an unfavorable interaction with negatively charged posttranslational modifications in CCR7. Here, we confirm this using a CCL19-variant lacking the basic C-terminus. This variant displays a 22-fold higher potency at CCR7 compared to WT CCL19 and is highly unaffected by the presence of C21TP. WT CCL19 has a short basic C-terminus, CCL21 a longer one. Here, we propose a way to differentially boost CCL19 and CCL21 activity as *short* and *long* versions of C21TP boost CCL19 activity, whereas only a long C21TP version can boost chemokines with a full-length CCL21 C-terminus.

## 1. Introduction

Chemokine receptors (CKRs) belong to class A G-protein-coupled receptors (GPCRs) [1]. CKR signaling involves several intracellular effectors, each of which is regulated in a stepwise manner. Upon ligand binding and receptor activation, CKRs induce G_αi_ signaling, β-arrestin recruitment, and internalization, leading to the chemotaxis of leukocytes [2]. The chemokine system is reckoned as highly promiscuous as some CKRs interact with multiple chemokines and some chemokines activate multiple receptors [2,3]. Chemokines that share the same receptor may give rise to differential changes in receptor conformation, resulting in the preferential activation of different signaling pathways by each ligand, a phenomenon known as ligand bias. CCL19 and CCL21 both interact with CCR7 but regulate different aspects of leukocyte behavior. CCL21 is the main driver of the lymph node (LN) homing of antigen-presenting cells (APCs), including dendritic cells (DCs), whereas CCL19 is responsible for the DC scanning mode that leads up to the priming and activation of naïve T-cells [4,5]. The CC-chemokines CCL19 and CCL21 induce biased signaling through their shared receptor CCR7 [6,7,8], although they display similar receptor affinities [9]. The underlying mechanism of this biased signaling between CCL19 and CCL21 is, however, not fully understood.

CCL19 provides a strong short-lived signal through G_αi_, and, subsequently, β-arrestin recruitment and internalization of CCR7. In contrast, CCL21 induces a weak persistent signal through G_αi_ while being a weak inducer of β-arrestin recruitment and internalization [2,7,10]. Furthermore, CCL19 is a strong chemotactic cue compared to CCL21 at 10 nM [8] and has been revealed to be 10–100-fold more potent than CCL21 [10].

While CCL19 and CCL21 have the same chemokine fold, they only share an amino acid identity of 32%, containing distinct N-termini and core domains. Furthermore, CCL21 contains a 37 amino acid (aa) extended positively charged C-terminus, not mirrored in CCL19 [11,12]. The mechanism by which the extended CCL21 C-terminus affects the structure and function of CCL21 is poorly understood. As opposed to other chemokines, the CCL21 C-terminus is unstructured and does not inaugurate a stable fold [13]. Previous studies have suggested that CCL21 adopts an autoinhibited conformation enforced by the extended C-terminus, which could be the cause for the lower potency of CCL21 [12,14]. Surprisingly, polysialylation, which is a rare posttranslational modification (PTM) occurring in DCs, where multiple polysialic acid residues (PolySia) are attached to glycan structures decorating the cell, appears to relieve CCL21 autoinhibition [14]. PolySia is attached directly to CCR7 and enhances the potency of CCL21 in activated DCs [14]. The extended CCL21 C-terminus is naturally cleaved off by DC-released proteases and plasmin, generating a much more potent ligand, i.e., tailless CCL21 (CCL21^tailless^ or soluble CCL21) [15,16]. In contrast to full-length CCL21, CCL21^tailless^ displays a strong chemotactic potential similar to CCL19 at 10 nM [8].

Similar to that observed by Ott et al. in 2006, we have previously revealed that the N-termini of CCL19 and CCL21 are interchangeable [17,18]. Interchanging the first 16 N-terminal amino acids between CCL19 and CCL21 did not significantly alter the potency of the chemokines, suggesting that the bias is determined by the core domains of the ligands and/or the extended basic C-terminus of CCL21. Transferring the C-terminus of CCL21 to CCL19 lowers the potency in G_αi_ signaling (CCL19^CCL21 C-term^), although not to the same level as CCL21, while removing the C-terminus from CCL21 increases the potency to a level closer to CCL19 (CCL21^tailless^) [11].

This indicates that the low potency of CCL21 results from a synergistic mechanism between the C-terminus and other elements of the protein. CCL19^CCL21 C-term^ encompasses a different N-terminus and a different chemokine core domain than CCL21, which may affect C-terminus positioning. By concomitant transfer of both the N- and C-terminus of CCL21 to CCL19, generating the chimeric chemokine variant CCL19^CCL21N-term|C-term^, we investigate the underlying mechanism for the low potency of CCL21 compared to CCL19 and if this, to some degree, can be credited the core domain or a combined effect of the CCL21 N- and C-terminus. We find that this transfer dramatically reduced the potency of CCL19^CCL21N-term|C-term^ and that the low potency of CCL21 compared to CCL19 is likely a synergistic effect of the N- and C-terminus of CCL21. Our findings indicate that CCL19^CCL21N-term|C-term^ engages CCR7 in a similar manner as CCL21, as alanine substitution of residue R209 in the extracellular loop 2 (ECL2) of CCR7 does not impair CCL19^CCL21N-term|C-term^ -induced G_αi_ signaling, which previously has been recognized to impair CCL19- but not CCL21-induced G_αi_ signaling and chemotaxis [18].

**Figure 1:**
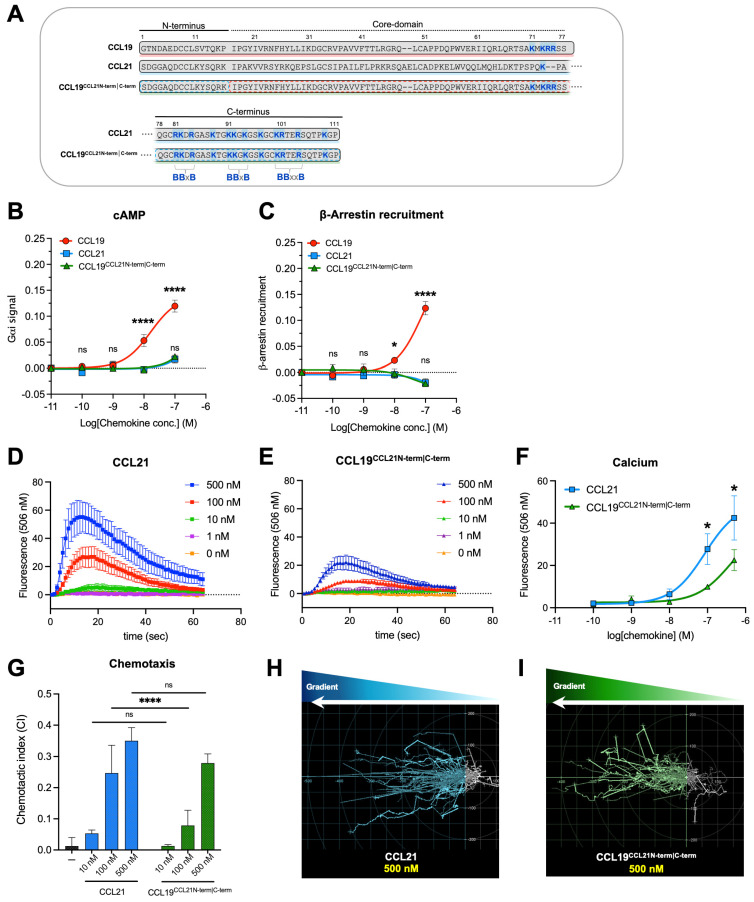
Signaling profiles of CCL19^CCL21N-term|C-term^, CCL21, and CCL19 in transfected cell lines and primary cells. (**A**) Alignment of CCL19, CCL21, and the chimeric chemokine CCL19^CCL21N-term|C-term^ encompassing the core domain of CCL19 (aa 17–77), the N-terminus (aa 1–16), and the C-terminus of CCL21 (aa 78–111). Basic residues in the C-terminal region of all three chemokines are written in bold and marked with blue, and the BBx(x)B motifs within CCL21 (3 motifs) and CCL19 (1 motif) are identified. As can be readily observed, the C-terminus of CCL19^CCL21N-term|C-term^ contains the 3 BBx(x)B motifs from CCL21, together with 1 BBx(x)B motif from CCL19, thus 4 BBx(x)B motifs in all. The signaling properties of CCL19^CCL21N-term|C-term^ in (**B**) G_αi_ signaling and (**C**) β-arrestin recruitment assay. G_αi_ signaling and β-arrestin recruitment were assessed using BRET-based assays. Statistical significance between dose–response curves was calculated using two-way ANOVA with Tukey’s multiple comparisons tests *(n =* 3–6). Calcium signal in DCs stimulated with (**D**) CCL21 or (**E**) CCL19^CCL21N-term|C-term^. Changes in intracellular calcium are measured by measuring the fluorescence intensity of the dye Fluo-4, which increases fluorescence emission upon calcium-binding. Data are background subtracted to show relative changes in calcium flux *(n* = 5*)*. (**F**) Dose–response curve of calcium signal in figure (**D**,**E**), where the maximal fluorescence value for each chemokine concentration is plotted. Statistical significance between dose–response curves was calculated using two-way ANOVA with Sidak’s multiple comparisons tests. (**G**) moDC chemotaxis and spider diagrams depicting the DC migration pattern towards the chemokine gradients; (**H**) CCL21 100 nM and (**I**) CCL19^CCL21N-term|C-term^ 100 nM. DC migration was assessed using time-lapse recordings (12 h) of moDCs. Statistical significance between bar graphs (CI values) was calculated using one-way ANOVA with Tukey’s correction for multiple tests. (*n* = 3). * *p* < 0.05, **** *p* < 0.0001, ns not significant.

We have previously hypothesized that O-glycosylations capped with sialic acids at the N-terminus of CCR7 interfere with the receptor docking of CCL21 and, to a lesser extent, CCL19 due to the clusters of basic residues in the C-terminal parts of both chemokines. CCL21 encompasses a long basic C-terminus with multiple basic amino acid (aa) stretches in the form of BBx(x)B domains (where B indicates a basic aa, and x can be any aa). CCL19 encodes a short basic C-terminus with only one such stretch (Figure 1A).

Interestingly, we recently published that the low potency of full-length CCL21 can be rescued by the addition of the free CCL21 C-terminal peptide C21TP, which can be both N- and C-terminally truncated while retaining its boosting ability (as long as the total number of BBx(x)B domains is 3 or more) [19]. The C21TPs were revealed to directly interact with the O-glycosylated CCR7 N-terminus, which may alter the receptor conformation and thus make the binding pocket more accessible to full-length CCL21 [19].

Here, we show that CCL19 potency is enhanced with both short (2 BBx(x)B) and long (3 or more BBx(x)B) variants of C21TP, in contrast to CCL21 potency that is enhanced only by the long variants. Supporting the aforementioned results, boosting the signaling of CCL19^CCL21N-term|C-term^ with short and long variants of C21TP revealed that CCL19^CCL21N-term|C-term^ bears more resemblance to CCL21 than CCL19, as it is boosted by long C21TP variants only. Likewise, both short and long variants of C21TP boosted the G_αi_ signaling of a semi-truncated CCL21 variant (encompassing aa 1–91) that, like CCL19, harbors only one BBx(x)B domain. This indicates that ligands comprising a short C-terminus can be boosted to the same extent using long and short C21TP variants, whereas ligands that harbor a long basic C-terminus need longer C21TP variants for efficient boosting. As CCL19^CCL21N-term|C-term^ retains the poor potency exhibited by CCL21, the combined effect of the CCL21 N-terminus and the CCL21 C-terminus, similar to the combined effect of the CCL21 core domain and C-terminus, confer diminished chemokine activity. To establish a firm link between poor chemokine potency and the presence of basic residues in the chemokine C-terminus, we tested the signaling of a variant of CCL19, where all basic residues in its short C-terminus had been substituted with alanine. This variant displayed a 22-fold increase in potency in G_αi_ signaling compared to WT CCL19 and was only boosted to a minor extent by both short and long C21TP variants.

## 2. Results

### 2.1. The CCL21 N- and C-Terminus Cooperatively Reduce Potency

To investigate if the low potency of CCL21 compared to CCL19 is determined by the core domain or a combined effect of the N- and C-terminus, we concomitantly transferred the N- and C-terminus of CCL21 to CCL19, thereby generating the chimeric chemokine variant CCL19^CCL21N-term|C-term^ (CCL21^1−16^-CCL19^17−77^-CCL21^78−111^), c.f. Figure 1A. As previously reported, both CCL19 and CCL21 signal through CCR7 and activate G_αi_ protein signaling and β-arrestin2 recruitment, with CCL21 displaying a much weaker potency and a restricted stimulation of arrestin recruitment [7,11]. The signaling properties of CCL19^CCL21N-term|C-term^ were examined in CHO-K1 cells using BRET-based assays to measure G_αi_ signaling and β-arrestin recruitment and tested in parallel with CCL19 and CCL21. Interestingly, transferring both the N- and C-terminus of CCL21 to CCL19 dramatically decreased the potency of CCL19^CCL21N-term|C-term^, resembling the response of CCL21 and overlaying the dose–response curves, with no significant differences in both G_αi_ signaling and β-arrestin recruitment (Figure 1B,C). We next sought to investigate if CCL21 and the chimeric CCL19^CCL21N-term|C-term^ also induce similar responses in a more biologically relevant setting. Using human blood monocyte-derived mature dendritic cells (moDCs), we investigated the ability of the two chemokines to induce changes in calcium flux in primary cells naturally expressing CCR7. Ex-vivo matured moDCs were loaded with the calcium-binding dye Fluo4, and real-time changes in calcium flux upon stimulation were recorded. Both CCL21 and the chimera CCL19^CCL21N-term|C-term^ induced calcium flux within half a minute in a dose-dependent manner (Figure 1D,E). The calcium response induced by CCL19^CCL21N-term|C-term^ was significantly lower than that induced by CCL21 (Figure 1F), indicating that these two ligands interact with CCR7 in a somewhat different manner. Finally, using moDCs, we compared the migration-inducing ability of both chemokines by employing the Ibidi^®^ 3D chemotaxis μ-slide setup. As anticipated, CCL21 possessed a weak chemotactic potential at 10 nM but reaches a significantly higher chemotactic potential at 100 and 500 nM, displaying a chemotactic index (CI) value of CI ~0.27–0.37 and ~0.34–0.39, respectively (Figure 1G). A similar pattern is seen for CCL19^CCL21N-term|C-term^, which was tested in parallel with CCL21. The chimera induced no migration at 10 nM and displayed CI values of 0.08–0.14 at 100 nM and 0.24–0.30 at 500 nM (Figure 1G), although not significantly different from that of CCL21 at 500 nM. In comparison, CCL19 presented a strong migration cue already at 10 nM [19].

In the assays using moDCs, CCL19^CCL21N-term|C-term^ was slightly less potent than CCL21, whereas the responses elicited by these chemokines were equal in assays using transfected CHO-k1 cells. Overall, in all assays, CCL19^CCL21N-term|C-term^ resembled CCL21 much more than CCL19; together, these data suggest that the CCL19/CCL21 bias observed at CCR7 is not determined by the core domain but is likely a synergistic effect of the CCL21 N- and C-terminus.

In order to further establish this, we decided to look into the docking of the three chemokines to CCR7.

### 2.2. CCL19 Is Dependent on R209 in CCR7 ECL2 for Proper Receptor Activation—CCL21 and CCL19^CCL21N-term|C-term^ Are Not

We have previously identified essential residues for CCL19 and CCL21 in CCR7 using in silico modeling and receptor mutagenesis [18]. We found that residue R209 in ECL2 is selectively important for the CCL19 activation of CCR7, whereas it is not important for CCL21-induced CCR7 activation. Thus, an alanine substitution of this residue impaired both CCL19-induced G_αi_ signaling and chemotaxis, whereas CCL21 displayed a minor increase in potency, suggesting that these chemokines are differentially dependent on this region of ECL-2 and employ distinct docking modes.

**Figure 2:**
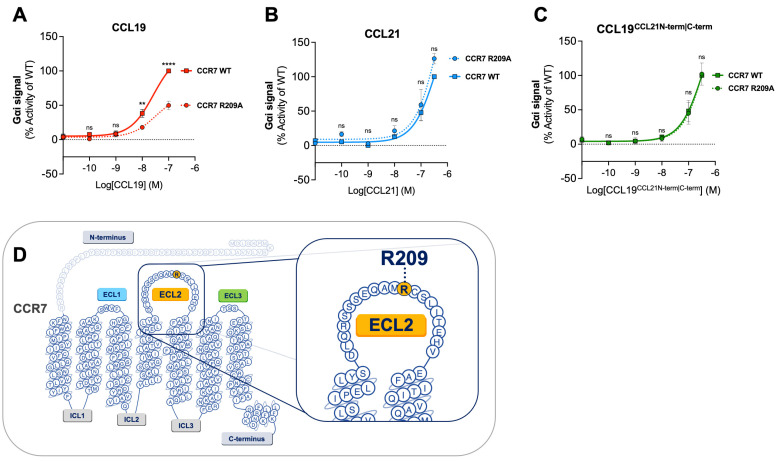
Alanine mutation of R209 in CCR7 does not affect CCL19^CCL21N-term|C-term^ potency in G_αi_ signaling. Dose–response curves of CCR7_WT_ and CCR7_R209A_ stimulated with (**A**) CCL19, (**B**) CCL21, and (**C**) CCL19^CCL21N-term|C-term^ in a cAMP accumulation assay. Each set of data has been normalized to the WT dose–response curve within each individual experiment before the collection of data. Significant differences between CCR7_WT_ and CCR7_R209A_ have been analyzed using two-way ANOVA with Šídák’s multiple comparisons test. ** *p* < 0.01, **** *p* < 0.0001, ns not significant. Data are presented as mean values ± SEM *(n = 3).* (**D**) Serpentine structure of CCR7 displaying the location of residue R209 in the ECL2.

Here, we examined the effect of the CCR7 R209A substitution on CCL19^CCL21N-term|C-term^-induced G_αi_ signaling to explore if our chimeric chemokine engages CCR7 in a manner resembling CCL19 or CCL21 (Figure 2A–C). CCL19^CCL21N-term|C-term^ was tested in parallel with CCL19 and CCL21. In accordance with previous data, CCL19-induced signaling at CCR7_R209A_ displayed a decreased activity compared to CCL19-induced signaling at CCR7_WT_ (Figure 2A). No significant increase or decrease in potency was observed with CCR7_R209A_ stimulated with CCL21 nor CCL19^CCL21N-term|C-term^ compared to CCR7_WT_ (Figure 2B,C). The alanine substitution of residue R209 does not negatively affect CCL19^CCL21N-term|C-term^-induced G_αi_ signaling, suggesting that CCL19^CCL21N-term|C-term^ engages CCR7 in a similar manner as CCL21.

### 2.3. The Chimeric Chemokine CCL19^CCL21N-term|C-term^ Resembles CCL21 with Regards to C21TP Boosting

As our chimeric chemokine, CCL19^CCL21N-term|C-term^, displayed a similar potency as CCL21 in the BRET-based assays and partly in the chemotaxis assay, except at 100 nM, we examined whether the signaling properties of CCL19^CCL21N-term|C-term^ were more reminiscent of CCL21 than CCL19 with regards to boosting the effect exerted by short and long C21TP variants.

**Figure 3:**
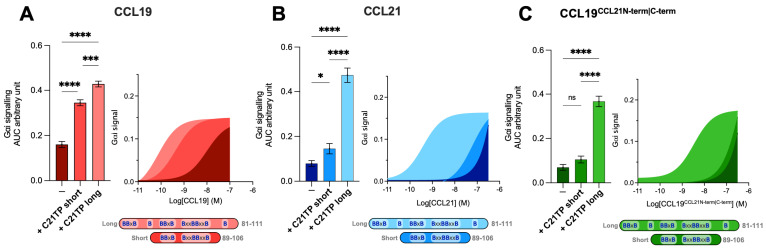
Long but not short C21TPs boost CCL19^CCL21N-term|C-term^ potency in G_αi_ signaling. Areas under the curve (AUCs, arbitrary units) of cAMP signaling are shown as bar graphs and AUC curves. The length and location of BBx(x)B motifs in the short and long C21TPs are shown as graphical bars. The effect of short (89–106) and long (81–111) C21TPs on G_αi_ signaling in response to (**A**) CCL19, (**B**) CCL21, and (**C**) CCL19^CCL21N-term|C-term^ was measured using BRET-based assays. C21TPs were added for a final concentration of 10 μM. Statistical significances were determined using one-way ANOVA with Tukey’s multiple comparisons test (*n* = 3). * *p* < 0.05, *** *p* < 0.001, **** *p* < 0.0001, *ns* non significant.

Prior to this, two variants of the C21TP, *long* (C21TP^81−111^) and *short* (C21TP^89−106^), were revealed to boost CCL19 and CCL21 activity differentially (Figure 3A,B). The long C21TP boosted both CCL19 and CCL21 to nearly the same extent, only with a slight difference in potency (Figure 3A,B), whereas the short C21TP only boosted CCL19-induced signaling, except for a small increase of CCL21 activity between 10–100 nM CCL21 (Figure 3B). Analyzing the activity of CCL19^CCL21N-term|C-term^ in the presence of either the long or the short C21TP revealed that CCL19^CCL21N-term|C-term^ -induced CCR7 activity, as observed with CCL21, could only be boosted in the presence of the long C21TP and not the short C21TP (Figure 3C).

However, the two chemokines were not boosted to the same extent by the long C21TP; thus, CCL19^CCL21N-term|C-term^ displayed a ~9-fold decrease in potency compared to CCL21 in the presence of this peptide (Supplementary Table S1). Likewise, the chimera CCL19^CCL21N-term|C-term^ displayed a 62-fold reduction in potency compared to CCL19 in the presence of the long C21TP (Supplementary Table S1). The concomitant transfer of the CCL21 N- and C-terminus to CCL19 synergistically contributes to reducing the potency of CCL19^CCL21N-term|C-term^, making it more reminiscent of CCL21 than CCL19, hence negatively affecting the potency of the ligand. Thus, the effect of transferring both the N- and C-terminus of CCL21 to CCL19 has a greater negative effect on CCL19 potency than the separate transfer of the CCL21 N- or C-terminus alone to CCL19 [12,18,19].

### 2.4. The Boosting Capacity of C21TPs Is Inversely Correlated with the Length of the Chemokine’s C-Terminus

**Figure 4:**
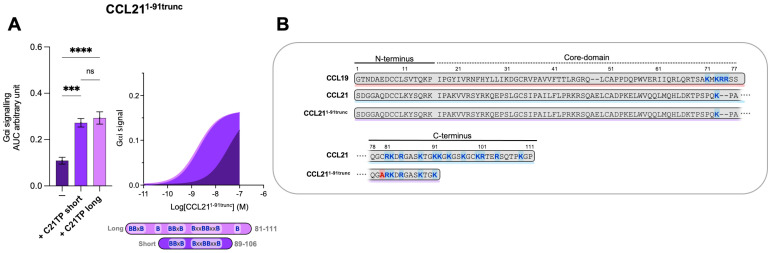
Long and short C21TP variants boost CCL21^1−91trunc^ activity in G_αi_ signaling equally. Areas under the curve (AUCs, arbitrary units) of cAMP signaling are shown as bar graphs and AUC curves. The length and location of BBx(x)B motifs in the short and long C21TPs are shown as graphical bars. (**A**) The effect of short (89–106) and long (81–111) C21TPs on G_αi_ signaling in response to CCL21^1−91trunc^ was measured using BRET-based assays. C21TPs were added to a final concentration of 10 μM. Statistical significances were determined using one-way ANOVA with Tukey’s multiple comparisons test (*n* = 3). *** *p* < 0.001, **** *p* < 0.0001, *ns* non significant. (**B**) Alignments of chemokine sequences; CCL19, CCL21, and CCL21^1−91trunc^. Basic residues in the C-terminal region of the chemokines are written in bold and marked with blue. The alanine substitution (C80A) is written in bold and marked with red.

It was surprising to observe that the short and long C21TPs exerted differential effects on CCL21- and CCL19-induced signaling. Given the different lengths of the C-terminal domains within these two chemokines, we decided to evaluate the effect on a semi-truncated CCL21. The semi-truncated CCL21 1–91 C80A (from now on referred to as CCL21^1−91trunc^) contains a C80A substitution to avoid alternative disulfide bonding between the free C80 (normally found in a disulfide with C99) and other cysteine residues in CCL21 or oxidative CCL21^1−91trunc^ dimer formation. CCL21^1−91trunc^ harbors only one BBxB domain in its C-terminus and, in that way, resembles CCL19 (Figure 4B). As previously touched upon, the truncated C21TP variants boost CCL19- and CCL21-induced signaling differentially.

To evaluate if the length and number of basic residues in the C21TPs dictate their ability to boost chemokines with a long versus short C-terminus, we examined the boosting capacity of the long C21TP and the short C21TP on CCL21^1−91trunc^. We found that in the presence of the long C21TP and the short C21TP, CCL21^1−91trunc^ displayed a ~19.7-fold and ~16.6-fold increase in potency, respectively (Table S1) (Figure 4A). CCL21^1−91trunc^ is more potent than CCL21 [12], a fact that is most likely due to the shorter basic C-terminus of this variant compared to the full-length CCL21 (Figure 4B). Overall, this suggests that ligands comprising a short basic C-terminus (i.e., CCL19 and CCL21^1−91trunc^) can be boosted to the same extent using both short and long C21TP variants, while ligands encompassing an extended basic C-terminus with multiple BBx(x)B domains (i.e., CCL21 and CCL19^CCL21N-term|C-term^) need long C21TP variants to reach a full-boosting effect.

### 2.5. C-Terminal Basic Residues in Chemokines Highly Influences Basal Potency and Determines the Boosting Ability of C21TPs

**Figure 5:**
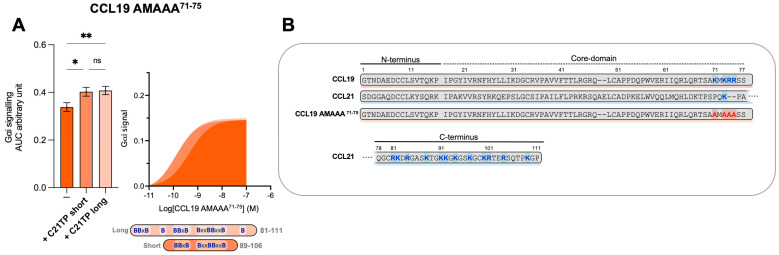
Removing C-terminal basic residues in CCL19 improves basal activity but does not boost activity in G_αi_ signaling. (**A**) Areas under the curve (AUCs, arbitrary units) of cAMP signaling are shown as bar graphs and AUC curves. The length and location of BBx(x)B motifs in the short and long C21TPs are shown as graphical bars. The effect of short (89–106) and long (81–111) C21TPs on G_αi_ signaling in response to CCL19 AMAAA^71−75^ was measured using BRET-based assays. C21TPs were added for a final concentration of 10 μM. Statistical significances were determined using one-way ANOVA with Tukey’s multiple comparisons test (*n* = 3). * *p* < 0.05, ** *p* < 0.01, *ns* non significant. (**B**) Alignments of chemokine sequences; CCL19, CCL21, and CCL19 AMAAA^71−75^. Basic residues in the C-terminal region of the chemokines are written in bold and marked with blue. The alanine substitutions K71A, K73A, R74A, and R75A are written in bold and marked with red.

We have previously shown that C21TPs potentiate both CCL19 and CCL21 but not the naturally truncated variant of CCL21, CCL21^Tailless^ [19]. In contrast to CCL21^Tailless^, CCL19 contains a cluster of basic residues in its short C-terminus (K71, K73, R74, and R75A), and, thus, CCL19 could be somewhat restricted (like CCL21) when binding to CCR7 due to electrostatic interactions with negatively charged PTMs at the CCR7 N-terminus. To examine if the C21TP-boosting potential is related to basic residues in the C-terminus of chemokines, we substituted all basic residues in the CCL19 C-terminus with alanines and generated CCL19 K71A K73A R74A R75A (CCL19 AMAAA^71−75^). We examined the boosting capacity of the long C21TP and the short C21TP on CCL19 AMAAA^71−75^. Removing the basic residues from the C-terminus increased the potency of CCL19 AMAAA^71−75^ by ~22-fold compared to WT CCL19 (Table S1). In the presence of the short and long C21TP, CCL19 AMAAA^71−75^ only displayed a ~3.5-fold and ∼3-fold increase in potency (Table S1) (Figure 5).

### 2.6. Host Defense Peptides Similar to Short C21TP Selectively Boosts CCL19, Indicating That Chemokine Boosting Is a Highly Physiological Relevant Mechanism

As discussed earlier, versions of C21TP could play a physiological role in potentiating DC chemotaxis inside narrow lymph capillaries where the local concentrations of C21TP could build up in front of the migrating DCs, released through the proteolytic cleavage of CCL21 secreted by the lymphatic endothelium [19]. To cement that basic peptide boosting of chemokine activity is likely to occur in vivo and that there might be a great deal of interplay between the innate and adaptive immune response, we tested the effect of the host defense peptide (HDP) histatin-1 on CCL19- and CCL21-induced CCR7 signaling. We chose histatin 1 as a representative of antimicrobial peptides (AMPs) found in saliva. Histatin-1, -3, and -5 are very similar in structure when it comes to the spacing and overall content of basic amino acids (Arg, Lys, and His) [20]. They are classic examples of AMPs that are found in high concentrations in tissues exposed to excessive bacterial loads (e.g., the oral cavity and gastrointestinal tract).

**Figure 6:**
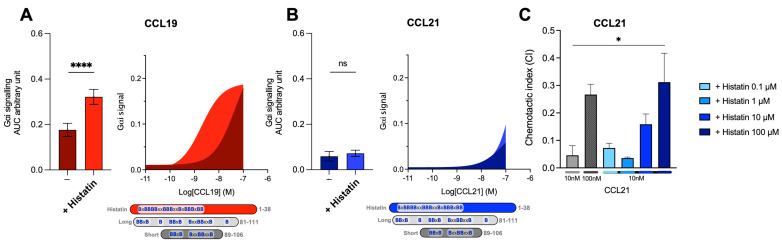
The human HDP histatin-1-like short C21TP displays differential boosting of CCL19 and CCL21. Areas under the curve (AUCs, arbitrary units) of cAMP signaling are shown as bar graphs and AUC curves. The effect of histatin-1 on G_αi_ signaling in response to (**A**) CCL19 and (**B**) CCL21 was measured using BRET-based assays. Histatin-1 was added for a final concentration of 10 μM. The length and location of BBx(x)B motifs in histatin-1 and the short and long versions of C21TP are shown as graphical bars. Statistical significances were determined using one-way ANOVA with Tukey’s multiple comparisons test (*n* = 4). **** *p* < 0.0001. moDC chemotaxis in response to (**C**) 10 and 100 nM CCL21 in presence of 0.1, 1, 10, and 100 μM histatine-1. DC migration was assessed using time-lapse recordings (12 h) of moDCs. Statistical significance between bar graphs (CI values) was calculated using one-way ANOVA with Tukey’s correction for multiple tests. (*n* = 3). * *p* < 0.05, **** *p* < 0.0001, *ns* non significant.

When aligning histatin-1 and the C21TP versions, the distribution of basic residues in histatin-1 carried similarities to both long and short C21TP. Histatin-1 is longer than the long C21TP version (81–111) but contains more centrally located basic residues, resembling the short C21TP (86–106) (Figure 6). Histatin-1 greatly increased CCL19 potency at CCR7 (Figure 6A), with no effect on CCL21 potency (Figure 6B). However, the addition of 10 μM histatin-1 displayed a minor boosting effect on CCL21-induced G_αi_-signaling at 100 nM CCL21 (Figure 6B). As CCL21 is the major LN homing chemokine, we examined if histatin-1 can boost CCL21 at higher concentrations. We tested DC chemotaxis towards 10 nM CCL21, using increasing amounts of histatin-1 (Figure 6C). Chemotaxis towards 100 nM of CCL21 was included as a positive control. We found that histatin-1 boosts CCL21-induced DC chemotaxis in a similar manner to what was recently published for long C21TP, although with lower potency [19].

## 3. Discussion

The inherent low potency of CCL21 compared to CCL19 has long been under investigation. In 2009, Bax et al. exposed how polysialylation (PolySia) expression by mature DCs, upregulated upon Toll-like receptor (TLR) engagement with damage-associated antigens (DAMPs), is required for the CCL21-directed migration of DCs. Especially, TLR4 engagement with LPS turned out to be a very potent stimulator of PolySia on pathogen-matured DCs [21]. The molecular requirements for this potentiation of CCL21 signaling turned out to be a direct engagement between CCL21 and PolySia, facilitating CCL21 capture to assist DC chemotaxis guidance by this chemokine. This study was followed up in 2016 by Kiermaier et al., revealing that PolySia is attached directly on CCR7 glycans and that this is required for efficient CCL21 but not CCL19- or CCL21^tailless^-induced DC chemotaxis in vivo [14]. Furthermore, this group revealed that the truncated version of CCL21, CCL21^tailless^, adopts a conformation that is different from CCL21, which led them to suggest an auto-inhibited model for full-length CCL21 where the C-terminus of CCL21 folds back and interacts with the core chemokine domain restraining the chemokine [14]. When CCL21 is incubated with free PolySia, the chemokine adopts a structure similar to CCL21^tailless^. Thus, PolySia, a rare modification that is restricted to neuronal tissue and mature DCs [22], appears to serve to not only capture but also to create a structure more fit for receptor activation. That the basic C-terminus of CCL21 lowers its potency was firmly established by Moussauras et al., who were able to show that the association of the C-terminus with the core chemokine is reminiscent of aa 80–90, whereas aa 92–100 confers low potency. The 92–100 stretch spans the region of the basic C-terminus harboring two BBxB domains [12]. Consensus now exists that the C-terminus of CCL21 confers low activity to CCL21. In 2018, we were able to show that the transfer of the C-terminus of CCL21 to CCL19 created a version of CCL19, CCL19^CCL21C-term^, that was less potent than CCL19 but still much more potent than CCL21 [11]. From this, we inferred that the C-terminus alone is not responsible for the poor potency of CCL21.

Another concept that was recently published by us is that the negative influence exerted by the long basic C-terminus of CCL21 on receptor docking can be relieved by the addition of excess free C-terminal peptides derived from CCL21, CCL21 tail-peptide, or C21TP. C21TP was found to interact directly with the CCR7 N-terminus with sialic-acid-capped O-glycosylation at position T38 [19]. Based on these data, we proposed a model predicting that interactions between the O-glycosylated CCR7 receptor N-terminus and the basic C-terminus of CCL21 normally abrogates the constructive docking of CCL21, offering an additional explanation to the inherently low potency of CCL21. The addition of excess free C21TP is predicted to shield the negative charges in the CCR7 N-terminus, counteracting unfavorable electrostatic interactions between the positively charged CCL21 C-terminus and the negative charges in the CCR7 N-terminus, in this way facilitating proper ligand docking. Complete removal of the basic C-terminus of CCL21, as found in CCL21^tailless^, creates a ligand that is in itself more potent than CCL21 and, at the same time, has lost its ability to be boosted by C21TP. To our surprise, CCL19, on its own a strong agonist of CCR7, could also be boosted by C21TP. Looking more carefully at the CCL19 sequence, we found that CCL19 too contains a basic C-terminus, although short (harboring one BBxB domain in comparison to the three, maybe four, partly overlapping domains found in the CCL21 C-terminus (Figure 1A), and thus its sensitivity to boosting by C21TP makes sense.

Based on studies from the 1990s, the classical model of chemokine receptor activation was proposed to follow the ‘two step, two site model’ describing how a chemokine reacts with its receptor in two ways: a temporal (two step) manner and a functional (two site) manner. During the first step, the chemokine core domain binds to the extracellular domain of the receptor, also called chemokine recognition site 1 (CRS1), ensuring high affinity without inducing receptor activation. Receptor activation occurs in the second step, where the chemokine N-terminus interacts with the seven-transmembrane (7TM) domain of the receptor through insertion into the receptor binding pockets (CRS2) [23,24,25]. Thus, as the ligand’s N-terminus, in general, is important for receptor activation, the different N-termini found in CCL19 versus CCL21 could also add to the difference in potency observed between these ligands.

CCR7 displays two electrostatic regions in its binding pockets, one involving TM3 and TM4 (major binding pocket; K137 and E193) and another involving TM1, TM2, and TM7 (minor binding pocket; K50, K51, R54, E118, and D309) [18]. The amino acid positions in this work are named according to the receptor start, including the 24 aa signal peptide, and were consistently 24 aa higher than the same positions when reported by Gaieb et al. [26]. In a structural analysis published by Gaieb et al. in 2016, they reported that CCL21 and CCL19, due to different N-termini, contact CCR7 in different ways. CCL21 has a more flexible (glycine-rich) and less negatively charged N-terminus (S^1^DGGGQD^7^CC in mice and S^1^DGGAQD^7^CC in humans), which probably allows it to contact both electrostatic regions within CCR7. In comparison, CCL19, due to a centrally positioned negatively charged D residue (G^1^ANDAED^7^CC in mice and G^1^TNDAED^7^CC in humans), cannot stretch across to contact both electrostatic regions in CCR7. Our data from 2016 confirms this and shows that CCL21 CCR7 engagement involves interaction with residues in both the major and minor binding pocket, whereas CCL19 engagement of CCR7 relies mainly on interactions with the minor binding pocket [18]. This forecasts that CCL21 may slide over both binding pockets and contact the major binding pocket from a different angle than CCL19. As proposed by our model presented in 2019, R209 in ECL2 (together with R54 in TM1) may form a lid that partly covers the entrance into the minor binding pocket of CCR7. This model predicts that this lid may guide the correct insertion of CCL19 into the binding pocket (creating a sort of directed entry) [11]. Despite the N-terminal differences described above, studies by Ott et al. [17] and by us [18] established that the N-termini of CCL19 and CCL21 are interchangeable. Thus, CCL19 with a CCL21 N-terminus (CCL19^CCL21Nterm^) remains potent, and CCL21 with a CCL19 N-terminus (CCL21^CCL19Nterm^) remains a poor agonist of CCR7, not fully cooperating with the influence of the differential N-termini on their own, on docking mode, as put forward by Gaieb. The C-terminus of CCL21 alone is not responsible for the low potency of CCL21, but it could, in principle, act in a synergistic manner with other parts of CCL21 that confer semi-low potency (e.g., CCL21 C-terminus) to effectively lower CCL21 ability to activate CCR7. Until now, two mechanisms describing how the C-terminus of CCL21 negatively affects ligand potency have been put forward, seemingly working side by side. (1) The C-terminus folds back upon the core domain of CCL21 to create an auto-inhibited version of CCL21, and (2) the C-terminus engages in electrostatic interactions with the CCR7 N-terminus, an interaction that negatively affects ligand docking mode.

To sum up the data presented on the native ligands of CCR7 and variants thereof, overall, CCR7 ligands fall into three groups based on the activation potential of CCR7. Ligands with no or very short basic C-termini are potent agonists (CCL19, CCL19^CCL21N-term^), and CCL21^tailless^, a ligand with CCL19 N-terminus and core domain combined with a long basic C-terminus, comprises a semi-potent agonist (CCL19^CCL21 C-term^); ligands with a CCL21 N-terminus and/or a CCL21 core domain combined with a long basic C-terminus comprise poor agonists (CCL21 and CCL21^CCL19N-term)^. In the current study, we set out to see whether the poor potency of CCL21 is conferred by the C-terminus in conjunction with the chemokine core domain (as represented by the two poor agonists identified above) or whether the same poor agonist function can be adopted by the concomitant transfer of both the CCL21 N-terminus and the CCL21 C-terminus. From the data obtained in this study, we establish that a ligand encompassing both the CCL21 N-terminus and the CCL21 C-terminus, but with the unrelated core domain of CCL19 (CCL19^CCL21N-term|C-term^), displays very low potency in Gα_i_ signaling, β-arrestin recruitment, and DC chemotaxis. Thus, it seems that poor potency can be conferred by auto-inhibition (dependent on the core domain and long basic C-terminus of CCL21) but also by the unfavorable docking mode enforced by electrostatic interactions between the basic C-terminus and the negatively charged CCR7 N-terminus (dependent on N-terminus and long basic C-terminus of CCL21). Thus, it is conceivable that the C-terminus forces the ligand to contact CCR7 in a mode similar to CCL21 and that this becomes extra problematic when the chemokine also contains the CCL21 N-terminus.

To investigate if the poor potency of CCL19^CCL21N-term|C-term^ relates to changes in the docking mode, we set out to see if the chimera relied on residue R209 in the CCR7 receptor extracellular loop 2 (ECL2), previously shown to be selectively important for CCL19, or whether its activity (like that of CCL21) is independent of this residue [18]. Whereas CCL19 relies on R209 for optimal receptor engagement and activation [18], using the R209A mutant version of CCR7, we confirm that CCL19^CCL21N-term|C-term^, like CCL21, is not dependent on this residue in ECL2 for proper receptor activation. As R209 is not important for CCL21 or CCL19^CCL21N-term|C-term^ activity, we again assume that CCL19^CCL21N-term|C-term^ takes after CCL21 more than CCL19 in the way it engages with CCR7.

**Figure 7:**
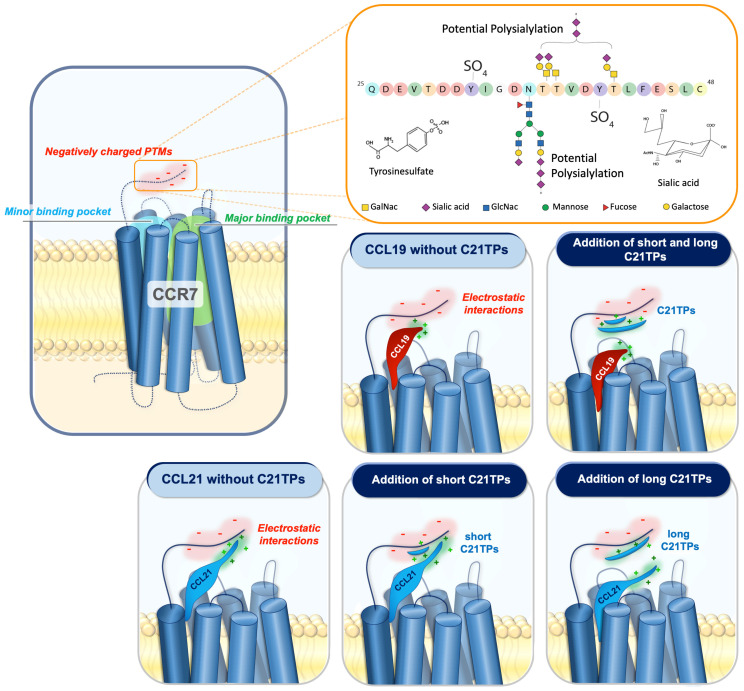
The addition of free CCL21 C-terminal peptides affects chemokine–receptor interaction.

To further establish if the basic C-terminus of CCL21, when attached to an unrelated core domain, creates docking problems relating to interference from electrostatic interactions with the negatively charged CCR7 N-terminus, we tested the effect of short and long variants of C21TP on the potency of CCL19, CCL21, and CCL19^CCL21N-term|C-term^. In the current study, we show that CCL19 can be boosted by both short and long C21TP variants, containing two versus three to four BBxB domains, whereas CCL21 and CCL19^CCL21N-term|C-term^ only gets boosted by the longer C21TP variants. Thus, ligands with an inherent low potency, conferred by a long basic C-terminus in combination with the flexible N-terminus of CCL21, can be rescued through the addition of positively charged peptides that shield negative charges in the CCR7 N-terminus to allow for a more favorable docking mode. Since CCL19, which encompass only a short basic C-terminus, can be boosted by shorter basic peptides, we propose that shorter peptides are sufficient to shield interactions between short positive chemokine C-termini and stretches of negative charges in CCR7, whereas longer peptides are needed to shield such interactions formed with chemokines encompassing longer basic C-termini. Apart from the O-glycosylation that we have found to underlie the boosting ability of C21TP at CCR7, CCR7 is known to harbor multiple residues in the receptor N-terminus that also decorate the receptor with negative charges in the surrounding area [22]. What modifications are important and relevant is a study in itself, and different modifications occur in different cell lines and primary cells depending on their repertoire of enzymes that carry out posttranslational modifications (PTMs). Additionally, such studies need careful consideration as there are several O-glycosylation sites; these may show heterogeneity in terms of both site occupancy and glycan structure in different cells and tissues. PTMs are often interdependent and can exert cross-talk [22]. This cross-talk is possibly due to changes in both spatial properties and the changed chemistry of a modified protein substrate compared to an unmodified substrate, both of which may affect enzymes that carry out PTMs [22]. Additionally, sialylation is a dynamic modification that can be remodeled by secreted or membrane-bound neuraminidases and sialyl-transferases [27,28]. Our model for how small versus long basic versions of C21TP support boosting is presented in Figure 7. It is conceivable that the interaction between the short basic C-terminus of CCL19 and negative charges in the CCR7 N-terminus can be prevented by short basic peptides (C21TP short), whereas chemokines with longer basic C-termini may need longer basic peptides (C21TP long) for shielding towards electrostatic interactions with the receptor N-terminus. That CCL19^CCL21N-term|C-term^, like CCL21, is only boosted by the long C21TP variant confirms that this chimera functionally behaves like CCL21. The C21TP peptide boosting effect happens on top of PolySia relief from auto-inhibition as signaling by ligands encompassing basic C-termini is boosted by C21TP in cell lines that are genetically manipulated to perform PolySia and in mature DCs (Supplementary Figure S1). Polysialylation may lead to the addition of up to 70 sialic acid residues attached to one glycan. It may seem contradictory that a single terminal sialic acid on CCR7 O-glycans seems to have a negative effect on docking of ligands with a basic C-terminus (as shown by us, as boosting with basic peptides is possible), whereas polysialylation has the opposite effect. This could possibly be explained by the longer polysialylations, rendering the bound chemokine more flexible during ligand docking into the receptor-binding pocket. However, it is also possible that polysialylation at multiple CCR7 glycans and/or neighboring substrates is contributing to the different outcomes. The physiological relevance of basic peptides and their cross-talk with chemokines were expanded upon by investigations into the boosting effect of the human defense peptide histatin-1. We found that histatin-1 boosts CCL19- and, to a lesser extent, CCL21-induced signaling through CCR7 and that this translates into the increased chemotaxis of activated human DCs towards CCL21 in the presence of histatin-1. Histatin-1 and other basic HDPs are part of our innate immune defense and partake in safeguarding our oral cavity. Although the oral mucosa forms a barrier that is thicker than the GI mucosa, the pharyngeal and periodontal epithelium that surround the teeth are weak. Thus, effective immune surveillance in the mouth is important. The saliva covering the mucosa contains immunoglobulins, such as secretory IgA, enzymes, and antimicrobial peptides (AMPs) such as histatins secreted by the salivary glands [20,29]. Apart from their direct antimicrobial effects, AMPs influence immune activation in various ways and, for that reason, are referred to as human defense peptides (HDPs). Many types of antigen-presenting cells, including Langerhans cells and various subsets of DCs, are present in the oral mucosa, submucosa, and mucosal/submucosal interface, where they survey the tissue and play important roles in balancing tolerance and inflammation [30,31,32]. Upon wounding/barrier breach and gingival infection, CCL19 and CCL21 expression in the oral tissue increases [33,34,35], guiding the re-localization of activated antigen-presenting cells from the periphery to lymphoid tissues. Under such circumstances, histatins in the saliva will come in contact with the underlying tissue and can interact with immune cells and molecules in the tissues as well as chemokine receptors on the DCs. Histatin-1 has been shown to increase the secretion of the antimicrobial skin and oral mucosa chemokine CCL20 [36], which has direct antimicrobial activity and acts as a chemoattractant of immature DCs and various other immune cell subsets [37]. Histatin-1 also stimulates endothelial cell adhesion, migration, and angiogenesis [38]. Our finding that histatin-1 boosts the action of the primary lymph node homing chemokine CCL21 is new and provides novel insights into a new mode of action of histatin-1 and underscores the physiological relevance of our findings on basic peptide boosting abilities.

Negative amino acids and/or posttranslational modifications (PTMs) such as sialylated and polysialylated N- and O-glycans and tyrosine sulfation at the N-terminus of CCR7 may affect the docking of CCL19 and CCL21 due to their positively charged C-terminal regions encompassing BBx(x)B motifs. Electrostatic interactions between the basic C-terminus of CCL19 and CCL21 and negatively charged PTMs in the CCR7 N-terminus restrain both chemokines from docking optimally into the receptor-binding pocket. CCL19 contains a short basic C-terminus with a few basic residues. The addition of both short and long C21TPs shields the negative charges in the CCR7 N-terminus enough to allow the proper receptor-docking of CCL19 and thus boosts signaling. In contrast to CCL19, CCL21 contains a long basic C-terminus, and thus the addition of short C21TPs does not shield the negative charges enough to prevent electrostatic interactions between the CCL21 C-terminus and the CCR7 N-terminus, whereas the addition of long C21TPs shields the negative charges sufficiently, allowing the optimal docking of CCL21 into the receptor-binding pocket, and thus boosts signaling. O-glycans are shown as core-1 structures at the acceptor sites previously reported [39] and N-glycan as a bi-antennary complex N-glycan at the consensus site. The two tyrosine sulfation sites have also been reported previously. Amino acids are colored according to the RasMol coloring scheme to illustrate the negative charges in the sequence.

In conclusion, the low ligand potency of CCL21 is conveyed by core and long basic C-terminus in combination or N-terminus and long basic C-terminus in combination, combinatory effects that can be separately transferred to other ligands through the transfer of either core domain and N-terminus or N- and C-terminus. Basic amino acid stretches in the unstructured C-terminus of chemokines in general lower ligand potency through a mechanism that involves electrostatic interactions between the positively charged C-terminus of the chemokine and the negatively charged receptor N-terminus (Figure 7). Such unfavorable interactions can be overcome by the addition of molar excess of basic peptides, which act to shield negative charges in the receptor N-terminus, consequently increasing ligand potency. Further, our data support that such boosting is likely to take place in vivo between host defense peptides (HDPs) expressed in the micromolar range and specific chemokines. As with C21TP, boosting ability relates to the number of basic domains in the HDPs and the length of the chemokine basic tail. This is likely to significantly increase the potency of selected chemokines, affecting immune cell localization to and within secondary lymphoid tissues and organs. Future studies will increase our understanding of how the innate immune system interplays with the adaptive immune system to control immune responses in different tissues with different HDP expression patterns.

## 4. Materials and Methods

### 4.1. Materials 

X-vivo 15 medium was from Lonza (Basel, Switzerland). Glucose, Human AB serum, Na_2_HCO_3_ (7.5%), MEM (10X), FBS, penicillin/streptomycin, glutamine, PGE2, and forskolin were from Sigma (St. Louis, MO, USA). IL-4, GM-CSF, TNF-α, IL-1β, and IL-6 were from Peprotech (Rocky Hill, NJ, USA). DMEM, RPMI, PBS, trypsin, and HBSS were from Thermo Scientific (Waltham, MA, USA). Lymphoprep was from STEMCELL Technologies (Vancouver, BC, Canada). PureCol Bovine Collagen I suspension was from Advanced Biomatrix (Carlsbad, CA, USA). Human CCL19 and CCL21 were purchased from R&D Systems (Minneopolis, MN, USA). CCL19^CCL21N-term|C-term^ (CCL21 residues 1–16, residues 17–77, CCL21 residues 78–111) was expressed and purified as described in detail for the chemokine CCL19 in Veldkamp et al. [40]. Coelenterazine was from Nanoligth (Pinetop, AZ, USA). Ibidi 3D chemotaxis slides were from Ibidi (Martinsried, Germany). C21TP variants were from Caslo (Lyngby, Denmark).

CCL21 1–91 C80A was produced as described in Moussouras et al. [12].

CCL19^CCL21N-term|C-term^ has the sequence below, where CCL21-derived aa are in normal text, and CCL19-derived aa are in italic and underlined (containing CCL21 residues 1–16, CCL19 residues 17–77, and CCL21 residues 78–111):

SDGGAQDCCLKYSQRKIPGYIVRNFHYLLIKDGCRVPAVVFTLRGRQLCAPPDQPWVERIIQRLQRTSAKMKRRSSQGCRKDRGASKTGKKGKGSKGCKRTERSQTPKGP

CCL19^CCL21N-term|C-term^ was purified as previously described for CCL21 [13]. Briefly, DNA coding for an SMT3- CCL19^CCL21N-term|C-term^ was cloned into pET28a and transformed into BL21(DE3) *E. coli*. One-liter cultures were grown at 37 °C in either lysogeny broth or [U-^15^N/^13^C] M9 minimal media to an optical density of 0.5–0.7 at 600 nm. Cultures were then induced with 1 mM isopropyl-β-D-thiogalactopyranoside (IPTG) for five hours. Cell pellets were collected by centrifugation and stored at −20 °C. Cells were resuspended in 10 mL of buffer A (50 mM sodium phosphate, 300 mM NaCl, 10 mM imidazole, pH 8.0) containing 1 mM phenylmethylsulfonyl fluoride (PMSF) and 0.1% (*v*/*v*) 2-mercaptoethanol. Resupended cells were lysed by sonication, which was followed by centrifugation at 15,000× *g* for 15 min. The pelleted inclusion body containing the His_6_-SMT3- CCL19^CCL21N-term|C-term^ was dissolved in 10 mL of buffer AD (50 mM sodium phosphate, 300 mM NaCl, 10 mM imidazole, and 6 M guanidine hydrochloride, 0.1% (*v*/*v*) 2-mercaptoethanol, pH 8.0), clarified by centrifugation at 15,000× *g* for 15 min and batch-loaded onto 2 mL of His60 nickel resin for thirty minutes. The column was washed with 40 mL of buffer AD and eluted with 30 mL of buffer BD (100 mM sodium acetate, 300 mM NaCl, 10 mM imidazole, and 6 M guanidine hydrochloride, pH 4.5). Elutions were dialyzed against 4 L of 0.3% acetic acid at 4 °C overnight, followed by dialysis against 4 L of 20 mM TRIS at pH 8.0. Ubiquitin-like protease 1 was added to the dialysate, followed by dialysis against a fresh 4 L of 20 mM TRIS at pH 8.0. The His_6_-SMT3 and CCL19^CCL21N-term|C-term^ in the dialysate were separated using cation exchange chromatography (HiTrap SP FF, binding and wash buffers were 100 mM Tris pH 8.0 with 50 mM NaCl, while the elution buffer was 100 mM Tris pH 8.0 with 2 M NaCl). CCL19^CCL21N-term|C-term^ was further purified using reverse-phase HPLC (0.1% aqueous trifluoroacetic acid buffer with a CH_3_CN gradient from 21% to 42% (*v*/*v*) over 30 min). CCL19^CCL21N-term|C-term^ identity and folding were confirmed using mass spectrometry and one- and two-dimensional protein NMR similar to previous descriptions [41].

CCL19 AMAAA^71−75^ has the sequence below (CCL19 containing K71A, K73A, R74A, and R75A substitutions, marked in italic and underlined):

GTNDAEDCCLSVTQKPIPGVIVRNFHYLLIKDGCRVPAVVFTTLRGRGLCAPPDQPWVERIIQRLQRTSA*A*M*AAA*SS.

Briefly, DNA coding for a CCL19 AMAAA^71−75^ was cloned into pET28a and transformed into BL21(DE3) *E. coli*. One-liter cultures were grown at 37 °C in lysogeny broth to an optical density of 0.8 at 600 nm. Cultures were then induced with 1 mM isopropyl-β-D-thiogalactopyranoside (IPTG) for five hours. Cell pellets were collected by centrifugation and stored at -20 °C. Cells were resuspended in 10 mL per liter of buffer A (50 mM sodium phosphate, 300 mM NaCl, 10 mM imidazole, pH 8.0) containing 1 mM phenylmethylsulfonyl fluoride (PMSF) and 0.1% (*v*/*v*) 2-mercaptoethanol. Resuspended cells were lysed by sonication, which was followed by centrifugation at 15,000× *g* for 15 min. The pelleted inclusion body containing the His_6_-SMT3- CCL19 AMAAA^71−75^ was dissolved in 20 mL of buffer AD (50 mM sodium phosphate, 300 mM NaCl 10 mM imidazole, and 6 M guanidine hydrochloride, 0.1% (*v*/*v*) 2-mercaptoethanol, pH 8.0), clarified by centrifugation at 15,000× *g* for 15 min and batch-loaded onto 4 mL of His60 nickel resin for 30 minutes. The column was washed with 40 mL of buffer AD and eluted with 30 mL of buffer BD (100 mM sodium acetate, 300 mM NaCl 10 mM imidazole, and 6 M guanidine hydrochloride, pH 4.5). CCL19 AMAAA^71−75^ was refolded by infinitely diluting the elution fraction (40 mL) into 240 mL of 100 mM TRIS at pH 8.0. The refolding mixture was concentrated to 50 mL and diluted 4-fold using 100 mM TRIS pH 8.0. Ubiquitin-like protease 1 was added, and the fusion protein was incubated overnight at room temperature. The His_6_-SMT3 and CCL19 AMAAA^71−75^ were separated using cation exchange chromatography (HiTrap SP FF, binding and wash buffers were 100 mM Tris pH 8.0 with 50 mM NaCl, while the elution buffer was 100 mM Tris pH 8.0 with 2 M NaCl). CCL19 AMAAA^71−75^ was further purified using reverse-phase HPLC (0.1% aqueous trifluoroacetic acid buffer with a CH_3_CN gradient from 30% to 60% (*v*/*v*) over 30 min). CCL19 AMAAA^71−75^ identity was confirmed using mass spectrometry [41].

The plasmid encoding CCR7 R209A was prepared from pcDNA with WT CCR7 using quick change PCR and primers holding the desired nucleotide changes, as described in Jørgensen et al. [11].

### 4.2. Methods

#### 4.2.1. Monocyte-Derived Dendritic Cell (moDC) Preparation

Buffy coats were obtained from Rigshospitalet, Copenhagen, as anonymous material and used in the studies, as approved by the local ethics committee. DCs were prepared from human peripheral blood mononuclear cells (PBMCs) isolated from buffy coats by centrifugation on a Lymphoprep gradient, as previously described [42]. Briefly, monocytes were isolated by plastic adherence of PBMC. Adhered monocytes were subsequently cultured and differentiated into immature DCs by incubation with IL-4 (250 U/mL) and GM-CSF (1000 U/mL) for 6 days, followed by activation into mature DCs by incubation with IL-6 (1000 U/mL), IL-1β (1000 U/mL), TNF-α (1000 U/mL), and PGE2 (1 µg/mL) for an additional 2 days in the same medium. The mature DCs are subsequently frozen in aliquots and stored at 80 °C.

#### 4.2.2. Cell Culturing

Human DCs were cultured in X-vivo 15 media with 2% human AB serum and glutamine. Adherent CHO-K1 cells were grown in RPMI with 10% FBS and penicillin/streptomycin. Suspension PolySia cell lines (knock-in of the polysialylation enzyme ST8SIA4 into CHO cells) and matching suspension WT CHO cells were grown in a 1:1 mix of EX-cell CD CHO fusion media and Balanced CD CHO growth A medium supplemented with 2% glutamine and penicillin/streptomycin. All CHO cells were maintained in a humidified incubator at 37 °C with 5% CO_2_. For stable cell lines, the passage number did not exceed 40. CHO cells expressing PolySia were generated by KI of ST8SIA4 using CRISPR/Cas9, as previously described [43].

#### 4.2.3. BRET Measurements of Gαi Signaling and β-Arrestin Recruitment

Adherent CHO-K1 cells were seeded in 6-well plates (500,000 cells/well) and transiently transfected the following day using lipofectamine as the transfection agent, while suspension CHO cells were transfected using FectoPro (200,000 cells/well). For BRET measurements of Gαi signaling, cells were transfected with vectors encoding the human CCR7_WT_/CCR7_R209A_ and the CAMYEL sensor (cAMP sensor, YFP-Epac-RLuc). For BRET measurements of β-arrestin recruitment, cells were transfected with vectors encoding human CCR7_WT_ and vectors expressing a Renilla luciferase arrestin3 fusion protein (RLuc8-Arr3) and a membrane-SH3-citrine protein (MEM-citrine-SH3). Lipofectamine transfections were terminated after 5 h by replacing the transfection media fresh medium. FectaPro transfections were terminated after 3 h by adding FectoPro booster and fresh media. Cells were resuspended in PBS w/glucose and subsequently aliquoted in white 96-well iso plates (~25,000 cells/well). When using C21TP variants, these were added to a final concentration of 10 μM/well, while an equal amount of PBS was added to control cells. The bioluminescence substrate coelenterazine was added to a final conc. of 5 µM. After 10 min, varying ligand concentrations were added. For BRET measurements of Gαi signaling, forskolin was added 5 min after the addition of the ligand to a final conc. of 5 µM. The plates were kept in the dark at all times. A Perkin Almer Envision machine was used for measuring the emission signals at 530 and 480 nm. The BRET signal was determined as the ratio eYFP(530 nm)/Rluc(480 nm).

#### 4.2.4. Calcium Signaling 

Pre-frozen DCs were thawed in X-vivo with 2% human serum and glutamine, then seeded (80,000 cells/well) in a black poly-D-lysine-coated 96-well plate with a clear bottom. After incubating for approximately 2 h, the medium was removed and the cells were incubated in the dark at 37 °C for 1 h with HEPES (20 mM) buffered HBSS with 1 mM CaCl_2_, 1 mM MgCl2, 250 µL probenicid, and 0.4% Fluo-4. After a two-time washing step in an equivalent buffer without Fluo-4, the plate was transferred to a preheated (37 °C) Flex station. Here, automated pipetting of chemokines and real-time fluorescence measurement at 506 nm allowed the immediate detection of changes in the intracellular calcium levels. Data are shown as changes in fluorescence at 506 nm after the addition of chemokines at time 0.

#### 4.2.5. Three-Dimensional (3D) Chemotaxis

Chemotaxis assays were conducted as previously described [8]. Briefly, mature human moDCs were left to acclimatize in medium for 30 min at room temperature (RT) upon defrosting before assay start. DCs were seeded in Bovine Collagen I mixture, prepared by mixing 10 µL Na_2_HCO_3_ (7.5%), 20 µL MEM (10X), 150 µL PureCol, and 90 µL DCs dissolved in X-vivo 15 medium (2 × 10^6^ cells/mL). After incubation for 45 min in a humidified incubator at 37 °C (5% CO_2_), the source and sink reservoirs were filled according to the manufacturer’s instructions, and chemotaxis was tracked in a time-lapse microscope with a humidified temperature-controlled stage incubator for 12 h at a 2 min interval. Cell migration (approximately 20–40 cells per viewing field) was tracked using a commercial tracking program (Autozell) and subsequently analyzed to get a population-based chemotactic index (CI) value (MATLAB). CI is a measure of net translocation distance to the source relative to the total distance traveled and was thus calculated as the ratio of the distance traveled in the direction of the gradient over the total distance traveled and, therefore, is a conservative measure of the directedness of cell migration.

## Data Availability

The datasets generated during and/or analyzed during the current study are available from the corresponding author upon reasonable request.

## Supplementary Materials

The following are available online at https://www.mdpi.com/article/10.3390/ijms23031397/s1.

## References

[1] Griffith J.W., Sokol C.L., Luster A.D. (2014). Chemokines and Chemokine Receptors: Positioning Cells for Host Defense and Immunity. Annu. Rev. Immunol..

[2] Esteen A., Elarsen O., Ethiele S., Rosenkilde M.M. (2014). Biased and G Protein-Independent Signaling of Chemokine Receptors. Front. Immunol..

[3] Schall T.J., Proudfoot A.E.I. (2011). Overcoming hurdles in developing successful drugs targeting chemokine receptors. Nat. Rev. Immunol..

[4] Laufer J.M., Kindinger I., Artinger M., Pauli A., Legler D.F. (2019). CCR7 Is Recruited to the Immunological Synapse, Acts as Costimulatory Molecule and Drives LFA-1 Clustering for Efficient T Cell Adhesion Through ZAP70. Front. Immunol..

[5] Embgenbroich M., Burgdorf S. (2018). Current Concepts of Antigen Cross-Presentation. Front. Immunol..

[6] Hauser M.A., Legler D.F. (2016). Common and biased signaling pathways of the chemokine receptor CCR7 elicited by its ligands CCL19 and CCL21 in leukocytes. J. Leukoc. Biol..

[7] Kohout T.A., Nicholas S.L., Perry S.J., Reinhart G., Junger S., Struthers R.S. (2004). Differential desensitization, receptor phosphorylation, beta-arrestin recruitment, and ERK1/2 activation by the two endogenous ligands for the CC chemokine receptor. J. Biol. Chem..

[8] Hjortø G.M., Larsen O., Steen A., Daugvilaite V., Berg C., Fares S., Hansen M., Ali S., Rosenkilde M.M. (2016). Differential CCR7 Targeting in Dendritic Cells by Three Naturally Occurring CC-Chemokines. Front. Immunol..

[9] Yoshida R., Nagira M., Kitaura M., Imagawa N., Imai T., Yoshie O. (1998). Secondary Lymphoid-tissue Chemokine Is a Functional Ligand for the CC Chemokine Receptor CCR7. J. Biol. Chem..

[10] Ricart B.G., John B., Lee D., Hunter C.A., Hammer D.A. (2010). Dendritic Cells Distinguish Individual Chemokine Signals through CCR7 and CXCR4. J. Immunol..

[11] Jørgensen A.S., Adogamhe P.E., Laufer J.M., Legler D.F., Veldkamp C.T., Rosenkilde M.M., Hjortø G.M. (2018). CCL19 with CCL21-tail displays enhanced glycosaminoglycan binding with retained chemotactic potency in dendritic cells. J. Leukoc. Biol..

[12] Moussouras N.A., Hjortø G.M., Peterson F.C., Szpakowska M., Chevigné A., Rosenkilde M.M., Volkman B.F., Dwinell M.B. (2020). Structural Features of an Extended C-Terminal Tail Modulate the Function of the Chemokine CCL21. Biochemistry.

[13] Love M., Sandberg J.L., Ziarek J.J., Gerarden K.P., Rode R.R., Jensen D.R., McCaslin D.R., Peterson F.C., Veldkamp C.T. (2012). Solution Structure of CCL21 and Identification of a Putative CCR7 Binding Site. Biochemistry.

[14] Kiermaier E., Moussion C., Veldkamp C.T., Gerardy-Schahn R., de Vries I., Williams L.G., Chaffee G.R., Phillips A.J., Freiberger F., Imre R. (2016). Polysialylation controls dendritic cell trafficking by regulating chemokine recognition. Science.

[15] Lorenz N., Loef E.J., Kelch I.D., Verdon D.J., Black M.M., Middleditch M.J., Greenwood D.R., Graham E.S., Brooks A.E., Dunbar P.R. (2016). Plasmin and regulators of plasmin activity control the migratory capacity and adhesion of human T cells and dendritic cells by regulating cleavage of the chemokine CCL21. Immunol. Cell Biol..

[16] Schumann K., Lämmermann T., Bruckner M., Legler D.F., Polleux J., Spatz J.P., Schuler G., Forster R., Lutz M.B., Sorokin L. (2010). Immobilized Chemokine Fields and Soluble Chemokine Gradients Cooperatively Shape Migration Patterns of Dendritic Cells. Immunity.

[17] Ott T.R., Lio F.M., Olshefski D., Liu X.-J., Ling N., Struthers R.S. (2006). The N-terminal domain of CCL21 reconstitutes high affinity binding, G protein activation, and chemotactic activity, to the C-terminal domain of CCL19. Biochem. Biophys. Res. Commun..

[18] Jørgensen A.S., Larsen O., Allmen E.U.-V., Lückmann M., Legler D.F., Frimurer T.M., Veldkamp C.T., Hjortø G.M., Rosenkilde M.M. (2019). Biased Signaling of CCL21 and CCL19 Does Not Rely on N-Terminal Differences, but Markedly on the Chemokine Core Domains and Extracellular Loop 2 of CCR7. Front. Immunol..

[19] Jørgensen A.S., Brandum E.P., Mikkelsen J.M., Orfin K.A., Boilesen D.R., Egerod K.L., Moussouras N.A., Vilhardt F., Kalinski P., Basse P. (2021). The C-terminal peptide of CCL21 drastically augments CCL21 activity through the dendritic cell lymph node homing receptor CCR7 by interaction with the receptor N-terminus. Cell. Mol. Life Sci..

[20] Sugiyama K., Ogino T., Ogata K. (1990). Rapid purification and characterization of histatins (histidine-rich polypeptides) from human whole saliva. Arch. Oral Biol..

[21] Bax M., van Vliet S.J., Litjens M., García-Vallejo J.J., van Kooyk Y. (2009). Interaction of Polysialic Acid with CCL21 Regulates the Migratory Capacity of Human Dendritic Cells. PLoS ONE.

[22] Goth C.K., Petäjä-Repo U.E., Rosenkilde M.M. (2020). G Protein-Coupled Receptors in the Sweet Spot: Glycosylation and other Post-translational Modifications. ACS Pharmacol. Transl. Sci..

[23] Kleist A.B., Getschman A.E., Ziarek J.J., Nevins A.M., Gauthier P.-A., Chevigné A., Szpakowska M., Volkman B.F. (2016). New paradigms in chemokine receptor signal transduction: Moving beyond the two-site model. Biochem. Pharmacol..

[24] Sanchez J., Huma Z.E., Lane J.R., Liu X., Bridgford J.L., Payne R.J., Canals M., Stone M.J. (2019). Evaluation and extension of the two-site, two-step model for binding and activation of the chemokine receptor. J. Biol. Chem..

[25] Thiele S., Rosenkilde M.M. (2014). Interaction of chemokines with their receptors--from initial chemokine binding to receptor activating steps. Curr. Med. Chem..

[26] Gaieb Z., Lo D.D., Morikis D. (2016). Molecular Mechanism of Biased Ligand Conformational Changes in CC Chemokine Receptor. J. Chem. Inf. Model..

[27] Varki A., Schnaar R.L., Schauer R., Varki A., Cummings R.D., Esko J.D., Stanley P., Hart G.W., Aebi M. (2015). Sialic Acids. Essentials of Glycobiology [Internet].

[28] Glanz V.Y., Myasoedova V.A., Grechko A.V., Orekhov A.N. (2019). Trans-sialidase Associated with Atherosclerosis: Defining the Identity of a Key Enzyme Involved in the Pathology. Curr. Drug Targets.

[29] Oppenheim F.G., Xu T., McMillian F.M., Levitz S.M., Diamond R.D., Offner G., Troxler R.F. (1988). Histatins, a novel family of histidine-rich proteins in human parotid secretion. Isolation, characterization, primary structure, and fungistatic effects on Candida albicans. J. Biol. Chem..

[30] Wu R.-Q., Zhang D., Tu E., Chen Q.-M., Chen W. (2014). The mucosal immune system in the oral cavity—an orchestra of T cell diversity. Int. J. Oral Sci..

[31] Hovav A.-H. (2013). Dendritic cells of the oral mucosa. Mucosal Immunol..

[32] Sharawi H., Heyman O., Mizraji G., Horev Y., Laviv A., Shapira L., Yona S., Hovav A., Wilensky A. (2021). The Prevalence of Gingival Dendritic Cell Subsets in Periodontal Patients. J. Dent. Res..

[33] McGrory K., Flaitz C.M., Klein J.R. (2004). Chemokine changes during oral wound healing. Biochem. Biophys. Res. Commun..

[34] Nassar M., Tabib Y., Capucha T., Mizraji G., Nir T., Saba F., Salameh R., Eli-Berchoer L., Wilensky A., Burstyn-Cohen T. (2018). Multiple Regulatory Levels of Growth Arrest-Specific 6 in Mucosal Immunity Against an Oral Pathogen. Front. Immunol..

[35] Song J.-H., Kim J.-I., Kwon H.-J., Shim D.-H., Parajuli N., Cuburu N., Czerkinsky C., Kweon M.-N. (2009). CCR7-CCL19/CCL21-regulated dendritic cells are responsible for effectiveness of sublingual vaccination. J. Immunol..

[36] Boink M.A., Roffel S., Nazmi K., Bolscher J.G.M., Veerman E.C.I., Gibbs S. (2017). Saliva-Derived Host Defense Peptides Histatin1 and LL-37 Increase Secretion of Antimicrobial Skin and Oral Mucosa Chemokine CCL20 in an IL-1α-Independent Manner. J. Immunol. Res..

[37] Dieu M.-C., Vanbervliet B., Vicari A., Bridon J.-M., Oldham E., Aït-Yahia S., Brière F., Zlotnik A., Lebecque S., Caux C. (1998). Selective Recruitment of Immature and Mature Dendritic Cells by Distinct Chemokines Expressed in Different Anatomic Sites. J. Exp. Med..

[38] Torres P., Díaz J., Arce M., Silva P., Mendoza P., Lois P., Molina-Berríos A., Owen G.I., Palma V., Torres V.A. (2017). The salivary peptide histatin-1 promotes endothelial cell adhesion, migration, and angiogenesis. FASEB J..

[39] Yang W., Ao M., Hu Y., Li Q.K., Zhang H. (2018). Mapping the O-glycoproteome using site-specific extraction of O-linked glycopeptides (EXo, O). Mol. Syst. Biol..

[40] Veldkamp C.T., Kiermaier E., Gabel-Eissens S.J., Gillitzer M.L., Lippner D.R., Di Silvio F.A., Mueller C.J., Wantuch P.L., Chaffee G.R., Famiglietti M.W. (2015). Solution Structure of CCL19 and Identification of Overlapping CCR7 and PSGL-1 Binding Sites. Biochemistry.

[41] Veldkamp C.T., Koplinski C.A., Jensen D.R., Peterson F.C., Smits K.M., Smith B.L., Johnson S.K., Lettieri C., Buchholz W.G., Solheim J.C. (2016). Production of Recombinant Chemokines and Validation of Refolding. Methods Enzymol..

[42] Hansen M., Met Ö., Larsen N.B., Rosenkilde M.M., Andersen M.H., Svane I.M., Hjortø G.M. (2016). Autocrine CCL19 blocks dendritic cell migration toward weak gradients of CCL21. Cytotherapy.

[43] Yang Z., Wang S., Halim A., Schulz M.A., Frödin M., Rahman S.H., Vester-Christensen M.B., Behrens C., Kristensen C., Vakhrushev S. (2015). Engineered CHO cells for production of diverse, homogeneous glycoproteins. Nat. Biotechnol..

