# Supplementary material for: Selective Boosting of CCR7-Acting Chemokines; Short Peptides Boost Chemokines with Short Basic Tails, Longer Peptides Boost Chemokines with Long Basic Tails
Source: Int J Mol Sci. 2022 Jan 26;23(3):1397. doi: 10.3390/ijms23031397 (PMC8836243; doi:10.3390/ijms23031397)
Supplement: Supplementary file 1 [file ijms-23-01397-s001.zip › ijms-1508478-supplementary.pdf]

Supplementary Material

Table S1. The effect of C21TPs on chemokine-induced $G_{\alpha i}$ signaling in CHO-K1 cells.

	None		Short C21TP		Long C21TP	
	EC_{50} (nM)	$pEC_{50} \pm$ SEM (M)	EC_{50} (nM)	$pEC_{50} \pm$ SEM (M)	EC_{50} (nM)	$pEC_{50} \pm$ SEM (M)
**CCL19**	9.93	8.00 ± 0.10	0.43	9.37 ± 0.074	0.075	10.13 ± 0.08
**CCL21**	-	5.12 ± 2.11*	63.10	7.2 ± 0.202	0.39	9.41 ± 0.17
**CCL19$^{CCL21N\text{-}term \mid C\text{-}term}$**	-	3.45 ± 121*	523.60	6.28 ± 0.18	3.47	8.46 ± 0.16
**CCL21$^{1\text{-}91trunc}$**	34.87	7.46 ± 0.15	2.09	8.68 ± 0.12	1.77	8.75 ± 0.14
**CCL19$^{AMAAA71\text{-}75}$**	0.45	9.35 ± 0.11	0.13	9.89 ± 0.11	0.15	9.83 ± 0.11

*Ambiguous

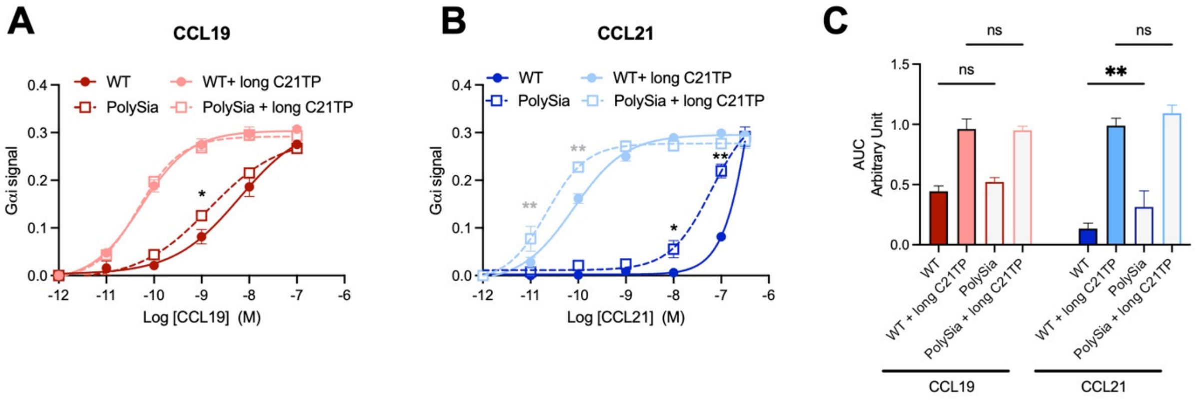

Figure S1. C21TP boosting takes place on top of polysialylation modifications: **A** and **B** cAMP signaling upon stimulation with CCL19 (A) or CCL21 (B) in WT cells or transgenic cells with knock in of the polysialylation (PolySia) enzyme ST8SIA4 measured using BRET-based assays. C21TP was added for a final concentration of 10 μM. Statistical significances were determined using two-way ANOVA with Tukey's multiple comparisons test (n = 3-4). *$P < 0.5$, **$P < 0.01$. **C** Area under the curve (AUC, arbitrary units) analysis of the dose-response curves presented in A and B. Statistical significances were determined using one-way ANOVA with Sidak's multiple comparisons test (n = 3-4). **$P < 0.01$, *ns* non significant.
